# Cisplatin liposome and 6-amino nicotinamide combination to overcome drug resistance in ovarian cancer cells

**DOI:** 10.18632/oncotarget.24708

**Published:** 2018-03-30

**Authors:** Daniela Catanzaro, Silvia Nicolosi, Veronica Cocetta, Marika Salvalaio, Andrea Pagetta, Eugenio Ragazzi, Monica Montopoli, Gianfranco Pasut

**Affiliations:** ^1^ Department of Pharmaceutical and Pharmacological Sciences, University of Padua, Padua, Italy; ^2^ Venetian Institute of Molecular Medicine, Padova, Italy; ^3^ Veneto Institute of Oncology IOV, IRCCS, Padua, Italy

**Keywords:** Cisplatin, drug resistance, drug delivery, liposomes, combination therapy

## Abstract

Ovarian cancer is an aggressive and lethal cancer usually treated by cytoreductive surgery followed by chemotherapy. Unfortunately, after an initial response, many patients relapse owing mainly to the development of resistance against the standard chemotherapy regime, carboplatin/paclitaxel, which is also affected by heavy side effects. In view to addressing such issues here, an association of liposomal cisplatin with 6-amino nicotinamide is investigated. It is known that resistant cells increase their demand for glucose, which is partially redirected toward the pentose phosphate pathway (PPP). Interestingly, we have found that also a cisplatin-resistant subclone of the ovarian cancer cells IGROV1 switch their metabolism toward the glycolytic pathway and rely on PPP to elude cisplatin cytotoxicity. The drug 6-amino nicotinamide, an inhibitor of the enzyme glucose-6-phosphate dehydrogenase (the rate-limiting step of the PPP) can restore the sensitivity of resistant cells to cisplatin. Then, to reduce the toxicity of cisplatin and prolong its action, a lyophilized stealth liposomal formulation of cisplatin was developed. The combination treatment of liposomal cisplatin and 6-amino nicotinamide showed promising cytotoxic activities in drug-resistant cells and a prolonged pharmacokinetics in rats, thus opening the way for a new therapeutic option against ovarian cancer.

## INTRODUCTION

Ovarian cancer (OC) is one of the most aggressive and lethal gynecological cancers with an incidence in 2012 of about 240,000 women/y and a mortality of about 150,000 women/y [1]. Nevertheless, the incidence and mortality forecasts of this tumor are expected to increase in the next years [1], and the 5-year survival rate is approximately 45%. One of the main issues of OC is that about 70% of patients are diagnosed with advanced disease (FIGO III/IV tumor stages), with several tumor nodes present and disseminated in the peritoneal cavity, thus impacting the success of treatment [2]. Furthermore, ascites can be a reservoir of aggressive cancer cells that facilitate the peritoneal dissemination. Consequently, any new approach of OC therapy should target various kinds of tumoral cells and/or tissues. Currently, standard initial management of advanced stages of OC is cytoreductive surgery [3], followed by a chemotherapy based on an association of carboplatin-paclitaxel [4–6]. Unfortunately, although an initial 70-80% response rate, most patients relapse due to development of diseases resistant to chemotherapy [7]. Carboplatin/paclitaxel therapy is limited by the risk of cumulative peripheral neuropathy [8]. Better results have been achieved in clinical trials with cisplatin when the doses used are doubled, but this approach is impractical due to the associated toxicity with a high-dose chemotherapy. In fact, anticancer drugs are usually low molecular weight molecules presenting poor bioavailability, low water solubility, non-specific body distribution and very modest cancer cell selectivity; thus they have several systemic toxicities with a consequent low patient compliance. It is therefore evident that unravelling the mechanisms causing chemoresistance is crucial for personalized therapy and the improvement of patients' long-term survival.

In order to develop innovative strategies enabling to limit not only the onset of cisplatin-resistance mechanisms but also to reduce its dosage in chemotherapy, we explored which pathways are exploited by human ovarian cancer cells resistant to cisplatin (IGROV1 Pt) to escape drug cytotoxicity. Cisplatin resistance is a multifactorial phenomenon, whose molecular mechanisms are still not completely understood. In our previous studies, we demonstrated that, among others, also deregulated metabolism might be involved in the onset of drug resistance revealing that cisplatin-resistant ovarian cancer cells (C13) underpin profound metabolic changes as compared with their sensitive counterpart (2008) [9, 10]. In particular, we observed that resistant cells increase their demand for glucose that is partially redirected toward the pentose phosphate pathway (PPP). Here we investigated the hypothesis that IGROV1 cisplatin-resistant cells might present a similarly altered phenotype that could be effectively targeted to restore drug sensitivity. Interestingly, we have found that IGROV1 Pt cells switch their metabolism toward the glycolytic pathway and rely on PPP to elude cisplatin cytotoxicity. Moreover, It has been demonstrated that the drug sensitivity could be restored by inhibiting the enzyme glucose-6-phosphate dehydrogenase (G6PDH), the rate-limiting step of the PPP, with 6-amino nicotinamide (6-AN) [10].

Given these promising data about the metabolic phenotype of IGROV1 PT cells, this work aimed to develop a strategy able to target PPP and, at the same time, to reduce cisplatin toxicity by proposing a new combination approach of 6-AN and liposomes loaded with cisplatin.

Cisplatin-loaded liposomes are a good strategy to reduce side effects and improve the antitumor efficacy of the drug [11, 12]. Currently, there are different platin-based formulations in clinical trials such as Lipoplatin, loaded with cisplatin [13], and Lipoxal, loaded with oxaliplatin [14, 15]. Most of these platin-loaded liposomes are based on saturated phospholipids, which yield slow rate of drug release with a consequent low antitumor efficacy *in vitro* but better liposome stability *in vivo* [16]. In fact, the lipid composition, and therefore the transition temperature of phospholipids, is a very important criterion for the development of a successful liposomal drug delivery system of anticancer drugs. The rate of cisplatin release can be increased by using unsaturated phospholipids and a reduced amount of cholesterol, thus increasing the fluidity and permeability of phospholipid bilayer [17]. In fact, in the case of OC, the liposomal formulation will be injected intraperitoneally and, therefore, it is relevant that the loaded drug is released with a favorable kinetic allowing the desired cytotoxic effect. On the other hand, such formulation might be less stable *in vivo*, but this issue can be circumvented through a polyethylene glycol (PEG) coating of the vesicles' surface, an approach known as PEGylation and that prolongs the pharmacokinetic profile of liposomes [18]. Usually, the polymer is anchored to the phospholipid bilayers by a distearoyl phosphoethanolamine (DSPE) covalently coupled to PEG, PEG-DSPE. The so-called stealth liposomes (SLs) outperformed the classic naked liposomes (CL) in terms of circulation half-life *in vivo* and this technology has been used in clinical practice for more than 20 years [19]. It has been demonstrated that the hydrophobic anchor, interacting with the phospholipid bilayer, is extremely relevant to maintain the PEG chain attached to the surface of liposomes [20], thus avoiding interaction with blood proteins that promote liposome clearance [21]. To strengthen such interaction and consequently gain a further stabilization of the PEG coating we recently proposed a specific PEG-dendron able to carry up to four DSPE molecules. Such PEG-dendron-(DSPE)_n_ derivative yielded liposomes with a prolonged pharmacokinetic *in vivo*, gaining a further extension of the circulation half-life of these coated liposomes with respect to SLs; they were called super-stealth liposomes (SSLs) [22].

Owing to the interesting features of SSLs, we here investigated the potential of such delivery system, prepared with unsaturated phospholipids as the main constituent, in overcoming OC resistance to cisplatin by testing *in vitro* a combination of liposomal cisplatin and 6-AN.

## RESULTS

### Preparation and characterization of cisplatin-loaded liposomes

**Figure 1:**
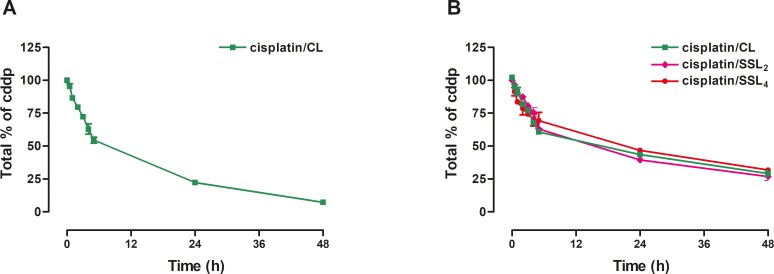
Cisplatin release from different liposomal formulations **(A)** Cisplatin release from CL liposomes prepared with only S75 and cholesterol (8:2 molar ratio). **(B)** Cisplatin release from CL, SSL_2_, and SSL_4_ liposomes prepared with S75:HSPC:Chol (63:25:12) for CL and of S75:HSPC:Chol:PEG-DSPE_2_ or PEG-DSPE_4_ (60:24:12:4) for SSL_2_ and SSL_4_, respectively. Data are the mean ± SEM of 3 independent experiments.

Initially, the CL liposomes were prepared by using only S75 and cholesterol (8:2 molar ratio). The hydrodynamic diameters of liposomes were approximately between 110-140 nm with a polydispersity index (PDI) of 0.1. This formulation showed a good encapsulation with an encapsulation efficiency of 18.5% ± 2.47 (0.85% cisplatin/lipid w/w). Cisplatin release studies of such formulation showed a very fast release of the encapsulated drug with almost 50% and 80% of cisplatin released in only 5 h and 24 h, respectively, (Figure 1A). To circumvent this issue about one-third of the phospholipids in the formulations was substituted with HSPC. These formulations presented a higher cisplatin encapsulation, as shown in Table 1, and a mean particle size comparable to previous formulations while, as desired, the cisplatin release was slower with 40-50% of the drug still entrapped in the liposomes at 24 h (Figure 1B). Although the drug release was optimized, the cisplatin release itself hampered the storage of the liposomes in the solution owing the accumulation of free cisplatin that would affect, the next *in vitro*/*in vivo* studies. Consequently, a lyophilized liposomal formulation was developed and the lyophilization process was optimized for such liposomes, thus allowing long-term storage without cisplatin release.

**Table 1:** Encapsulation efficiency and cisplatin loading of liposomal formulations (n = 3)

Formulation	cisplatin encapsulation efficiency (%)	cisplatin/lipid (% w/w)
cisplatin/CL	25.1 ± 0.71	1.14 ± 0.03
cisplatin/SSL_2_	25.7 ± 0.94	1.19 ± 0.04
cisplatin/SSL_4_	25.9 ± 0.86	1.21 ± 0.04

The liposomes were lyophilized and the effect of two disaccharides (trehalose and sucrose) as cryoprotectant was evaluated. Lyophilization can alter the organization of liposome bilayer causing vesicle disruption, with changes in vesicles size and PDI, and drug release. In this study, the changes of vesicles size and the PDI were measured before and after lyophilization by DLS. Different molar ratios of cryoprotectant with respect to phospholipids were tested by adding the disaccharide in the solution used for the hydration of the thin lipid film and in the purified liposome solution after the elimination of free cisplatin. The values of vesicles size and PDI of CL before and after lyophilization, are shown in Table 2. Lyophilization induced a dramatic increase on size and PDI values of liposomes when low amounts of cryoprotectant were used while a good protection was achieved for both cryoprotectants at the ratio 1:6 phospholipid/cryoprotectant (w/w). The lyophilization process did not affect the percentage of drug encapsulation into liposomes formulation. Similar results were obtained for SSL_2_ and SSL_4_.

**Table 2:** Investigation of different amounts of cryoprotectant for liposome stabilization during lyophilization (n = 3)

Molar ratio Phospholipids / Cryoprotectant	Mean Particle Size (nm ± SD)	PDI ± SD	Mean Particle Size (nm ± DS)	PDI ± SD
	Before lyophilisation		After lyophilisation	
**Sucrose**				
CL 1:2	136 ± 0.9	0.080 ± 0.009	557 ± 1.12	0.451 ± 0.052
CL 1:5	139± 0.8	0.098 ± 0.007	145 ± 1.2	0.141 ± 0.006
**Trehalose**				
CL 1:2	140.9 ± 1.6	0.098 ± 0.006	657 ± 1.52	0.651 ± 0.05
CL 1:5	120.4 ± 1.2	0.082 ± 0.022	235 ± 1.1	0.21 ± 0.06
CL 1:6	132.2 ± 1.1	0.062 ± 0.011	128 ± 1.15	0.082 ± 0.022

Sucrose was more effective in preserving the liposomes stability also at lower amounts with respect to trehalose. Although the interesting cryoprotective effect of sucrose, this disaccharide was affecting the *in vitro* cytotoxicity investigation because it induced cells growth and protected cisplatin-resistant cells against the activity of the drug (Supplementary Figure 1). Consequently, all the following studies have been performed with lyophilized liposomes containing trehalose as cryoprotectant at the molar ratio of 1:6 phospholipid/cryoprotectant (w/w).

Serum stability of CL, SSL_2_s, and SSL_4_s incubated up to 24 h in FBS/PBS (50/50) mixture, was evaluated by monitoring the size of vesicles and showed no significant changes of size and PDI during the time for all formulations.

### Glucose metabolism in cisplatin-resistant cells

**Figure 2:**
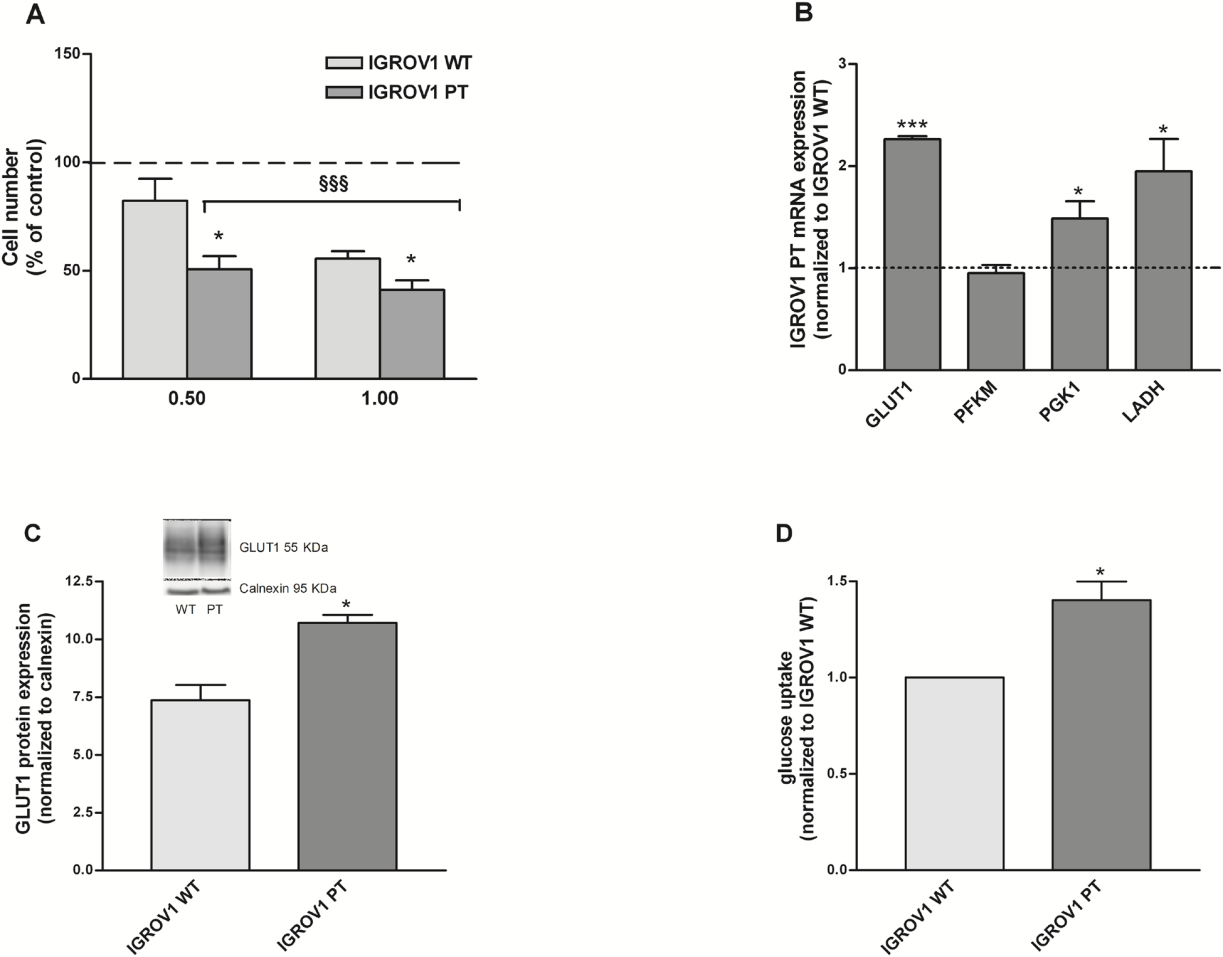
IGROV1 PT cells present an increased dependency on glucose **(A)** Cell viability after 24 hours of treatment with a glucose-free medium added with 0.5 and 1mM 2-DG. Data are expressed as the percentage of cell number compared to control. **(B)** Key genes mRNA levels involved in the glycolytic flux measured by qRT-PCR. All genes were normalized to calnexin as endogenous control and data are expressed as a ratio of IGROV1 PT to IGROV1 WT. **(C)** GLUT1 protein expression measured by western blotting. GLUT1 was normalized to calnexin. **(D)** Glucose uptake measured after incubation with the glucose analog 6-NBDG. Data are normalized to cisplatin-sensitive cells. Data are the mean ± SEM of at least 3-5 independent cultures. ^***^p<0.001, ^*^p<0.05*;* IGROV1 PT *vs* IGROV1 WT. §§§*p*<0.001*;* treatment *vs* control.

In line with our previous data, the cisplatin resistant cells exhibited a glucose-dependent phenotype [10]. In fact, the treatment with the glycolysis inhibitor 2-deoxyglucose (2DG) reduced cell viability of both cell lines and in particular that of resistant cells (−60%) (Figure 2A). mRNA expression of glycolysis enzymes was analyzed and as shown in Figure 2B, resistant cells presented higher mRNA levels of glycolytic enzymes compared to the wild type. In particular GLUT-1 mRNA resulted in 2.2 fold increase. The glucose transporter GLUT1 expression resulted up-regulated (1.33 fold) in resistant cells (Figure 2C). In line with these data, IGROV1 PT cells presented increased glucose uptake (Figure 2D). Together, these data showed that cisplatin-resistant cells are more dependent on glucose when compared to the cisplatin-sensitive counterpart.

### Glucose-6-Phosphate Dehydrogenase (G6PDH): Target for cisplatin-resistant cells

**Figure 3:**
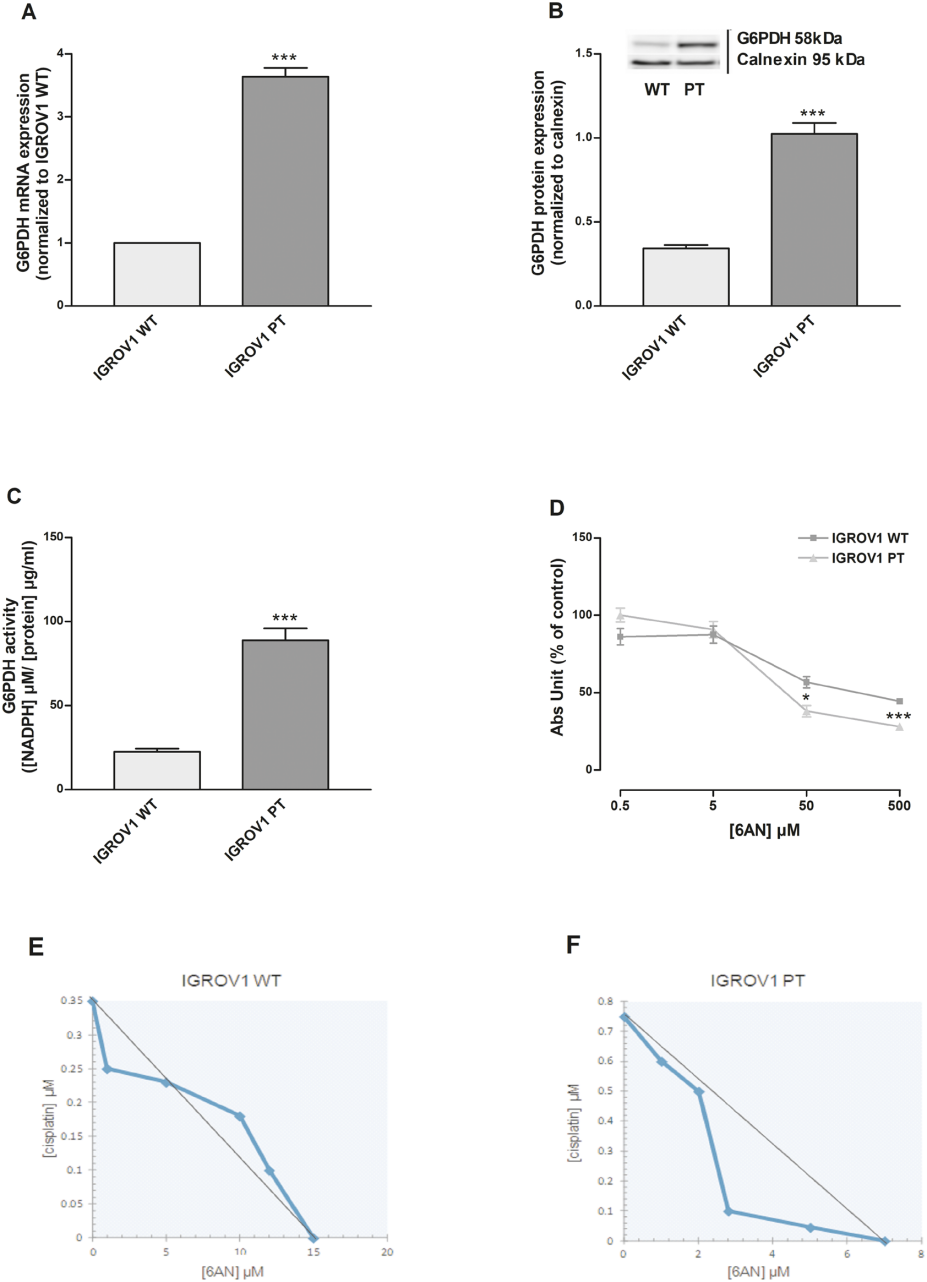
Inhibition of G6PDH, rate-limiting step of PPP, mainly affect cisplatin-resistant cells **(A-C)** G6PDH mRNA levels (A), protein expression (B) and enzymatic activity (C) were quantified respectively by qRT-PCR, western blotting and the enzymatic assay kit of Cayman Chemical Company. **(D)** Effect of 6AN (0.5-500 μM) on IGROV1 cell viability after 24+48 h of treatment. **(E-F)** Isobologram of cisplatin-sensitive (E) and cisplatin-resistant (F) cells showing the effect of 6-AN in association with cisplatin treatment. Data are expressed as a percentage of control. The graph was obtained using iso-effective drug concentrations causing 25% of the cytotoxic effect. Data are the mean ± SEM of at least 3-5 independent cultures. ^***^p<0.001, ^**^p<0.01, ^*^p<0.05; IGROV1 PT *vs* IGROV1 WT. +++*p*<0.001; treatment *vs* control.

G6PDH is a key enzyme of the PPP activity. G6PDH mRNA (Figure 3A) and protein (Figure 3B) expressions were increased in IGROV1 PT cells when compared to the sensitive counterpart (3.5 fold). Also, we evaluated the G6PDH activity, which was increased in IGROV1 PT cells in comparison to IGROV1 WT (Figure 3C). In order to confirm the specific phenotype, cells were incubated with a competitive G6PDH inhibitor: 6-AN [10]. As shown in Figure 3D, the cell viability was analyzed after 24h treatment with 6-AN (0.5-500 μM). The IGROV1 PT resistant cells, as compared to cisplatin-sensitive cells, were significantly more sensitive to 6-AN at the highest drug concentration. In order to verify the effects of the association of 6-AN with cisplatin at concentrations lower than its IC_50_ (Supplementary Table 3), isobolographic analysis (Figure 3E-3F) and correlated combination index (Supplementary Table 2) were calculated based on the data reported in Supplementary Table 1. In IGROV1 WT cells, the approximate linearity of the iso-effective concentrations (producing 25% of cytotoxic effect) suggests no interaction between the two drugs (Figure 3E). In the IGROV1 PT, a synergism was found (Figure 3F), but at higher concentrations of 6-AN. These results confirmed that cisplatin-resistant cells present a phenotype that has an increased dependence on glucose, is more sensitive to glycolysis inhibition and overexpressed PPP when compared to the cisplatin-sensitive counterpart.

### Cytotoxicity of liposomal cisplatin formulations

Cell viability assays of different cisplatin liposomal formulations (CL, SSL_2_, and SSL_4_) were carried out on sensitive and resistant cells. Empty liposomes did not show any cytotoxic effect in both cell lines at the concentrations used with the cisplatin loaded liposomes (supplementary material Supplementary Figure 2).

**Figure 4:**
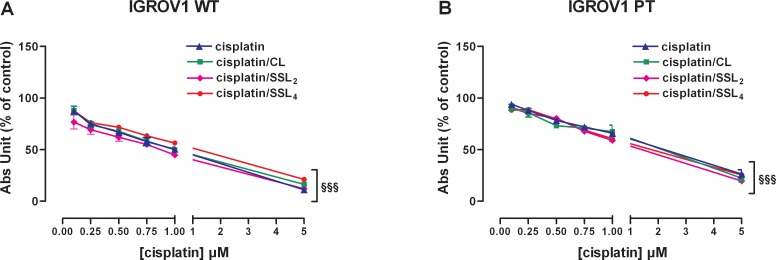
Cisplatin/liposomes delivery does not change cisplatin effectiveness in both IGROV1 cell lines **(A-B)** Effect of cisplatin alone or delivered by 3 different liposomal formulations (lyophilized with trehalose) in cisplatin-sensitive (A) and cisplatin-resistant (B) cells. The effect was measured after 24+48 h of treatments by SRB test. Data are the mean ± SEM of at least 3-4 independent cultures. §§§p<0.001; treatment *vs* control. Data are expressed as a percentage of control.

The effect on cell viability of cisplatin-loaded CLs, SSL_2_s, and SSL_4_s (0.1-5 μM cisplatin equiv.) after 24h treatment is shown in Figure 4. All liposomes presented similar concentration-dependent cytotoxic activity in both cell lines. These activities were also comparable to that of free cisplatin.

### Pharmacokinetics of cisplatin-loaded liposomes

**Figure 5:**
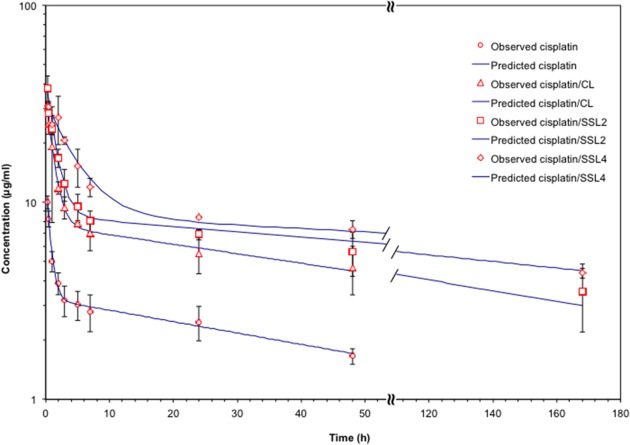
Liposomal formulations of cisplatin showed prolonged pharmacokinetic profiles Pharmacokinetic profiles of free cisplatin and cisplatin -loaded liposomes (CLs, SSL_2_s. and SSL_4_s) in female Lewis rats (n=3 per group) after i.v. injection via tail vein of 3 mg/kg cisplatin equiv. Data are the mean ± SEM.

The pharmacokinetic profiles of free cisplatin and cisplatin-loaded CLs, SSL_2_s, and SSL_4_s were investigated in female Lewis rats after a single bolus injection at 3 mg/kg cisplatin equiv. (Figure 5). The blood concentration of free cisplatin decreased rapidly after injection and the drug was not detectable after 48 h. As expected all liposomal formulations of cisplatin showed a prolongation in the pharmacokinetic profile with respect to the free drug. Both SSLs outperformed the CLs, in particular, SSL_4_s showed values of elimination half-life and area under the curve 2-fold and 3-fold higher than CLs (Table 3).

**Table 3:** Elimination half-life (T½ β) and area under the curve (AUC) of free cisplatin and liposomal formulations of cisplatin in Lewis rats (n=3) after the i.v. injection of 3 mg/kg of cisplatin equiv

Formulation	T½ β (h)	AUC (μg/ml·h)
cisplatin	52.14	250.19
cisplatin /CL	62.26	719.36
cisplatin /SSL_2_	110.72	1411.57
cisplatin /SSL_4_	179.81	2334.64

### Confocal microscope

**Figura 6:**
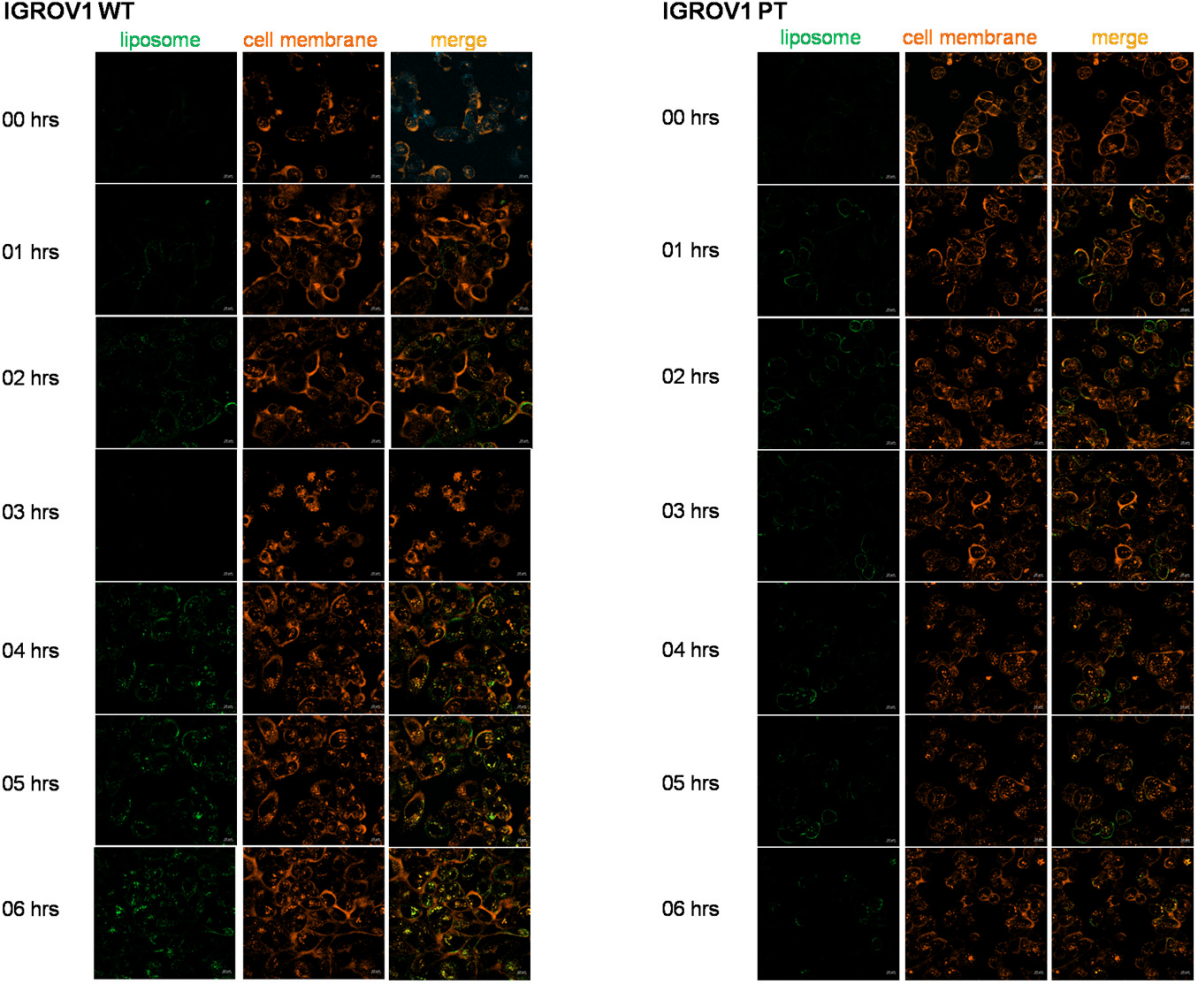
Time-lapse/live confocal laser scanning microscope images of IGROV1 WT and IGROV1 PT incubated with cisplatin/SSL_4_ Cells were incubated with fluorescein-labeled cisplatin/SSL_4_s 0.5 μM for 6 h. To stain plasma membrane in live cells CellMask^TM^ Orange Plasma membrane stain was used. Green: liposome, red: cell membrane. Scale bar is 10 μm.

Owing to the better pharmacokinetic performance of SSL_4_s, the next characterizations have taken into consideration only such formulation. Time-lapse/live confocal laser scanning microscope images (Figure 6) showed cellular internalization of fluorescein-labelled 0.5 μM cisplatin/SSL_4_s, following 6 h of treatment. The cell membrane of both sensitive and resistant cell lines was labeled with a CellMask^TM^ Orange Plasma membrane Stains. As shown in Figure 6, cisplatin/SSL_4_ interacted with cells and the fluorescence intensity was higher in wild-type with respect to resistant cell line. The fluorescence of fluorescein was co-localized with the membrane suggesting the fusion of the liposomes with the cells and the consequent release of cisplatin in the cytoplasm.

### Combination activity of cisplatin/SSL_4_ with 6-AN

**Figure 7:**
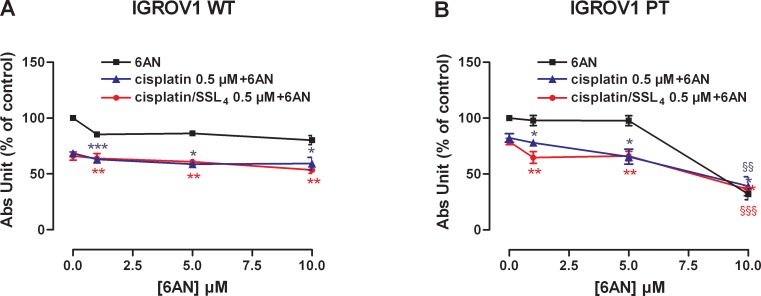
Co-treatment of 6AN and cisplatin/SSL4 increases drug activity in IGROV1 PT cells **(A-B)** Effect of 6AN and cisplatin/SSL_4_ association in cisplatin-sensitive (A) and cisplatin-resistant (B) cells. The effect was measured after 24+48 h of treatments by SRB test. Data are the mean ± SEM of at least 3-4 independent cultures. ^***^p<0.001, ^**^p<0.01, ^*^p<0.05; association *vs* 6AN. §§*p*<0.001, §§*p*<0.01; association *vs* cisplatin. Data are expressed as a percentage of control.

To evaluate the effect of G6PDH inhibition by the 6-AN and cytotoxic activity of cisplatin, in free or liposomal form, the two compounds were tested in IGROV1 WT and IGROV1 PT. The concentration of cisplatin equiv. was fixed at 0.5 μM, lower than cisplatin IC_50_ (reported in Supplementary Table 3), while increasing doses of 6-AN (1-10 μM) were tested (Figure 7). In IGROV1 WT the cell cytotoxicity was caused prevalently by cisplatin (free or SSL_4_). On the contrary, in resistant cells, the effect of the combination of 6-AN 1 μM with 0.5 μM cisplatin /SSL_4_ was more effective with respect to free cisplatin. These data demonstrated that the combination of cisplatin/SSL_4_ with inhibition of the PPP enzyme G6PDH can remarkably increase the cytotoxic activity of cisplatin and can overcome cisplatin cancer resistance. In Table 4 the combination index values calculated by the Chou & Talalay [23] are reported. The same combination was also tested in 2008 and C13 ovarian cancer cell lines confirming the efficacy of the treatment as reported in Supplementary Figure 5.

**Table 4:** Combination index of cisplatin/SSL_4_ and 6-AN in wild-type and resistant cell lines

Conc. cisplatin/SSL_4_ (μM)	Conc. 6AN (μM)	IGROV1 WT		IGROV1 PT	
		Effect	CI	Effect	CI
0.1	1	0.8845	7.371	0.873	0.956
0.1	5	0.7382	0.316	0.811	1.176
0.1	10	0.676	0.211	0.327	0.552
0.5	1	0.6397	0.852	0,647	0.661
0.5	5	0.608	0.717	0.661	1.147
0.5	10	0.535	0.487	0.362	0.681

## DISCUSSION

Chemotherapy remains the principal therapeutic approach in cancer treatment. Despite recent successes, resistance and relapse are still major obstacles [7]. Hence, novel pharmacological strategies addressing such issues are urgently needed.

Nanosized carriers have the potential to improve biopharmaceutical features, pharmacokinetic properties and the therapeutic effectiveness of anticancer drugs [24]. In the light of this view, we here report that a cisplatin liposomal formulation, obtained by a new approach of PEGylation, in combination with the PPP inhibitor 6-AN might represent a new therapy approach against cisplatin-resistant cancers.

Cisplatin is one of the most used chemotherapy drugs for the treatment of OC, but unfortunately, drug resistance is readily seen in patients during the therapeutic treatment [25]. Furthermore, the clinical use of cisplatin is limited by its severe side effects, including nephrotoxicity and ototoxicity. Dose-related and cumulative renal failure is the major dose-limiting toxicity of cisplatin. A single dose of 50 mg/m^2^ induced renal toxicity in 28% to 36% and ototoxicity in up to 31% of patients treated. The toxicity, also associated with drug resistance, created the limits in clinical management. Strategies for modifying cisplatin uptake into cancer cells and the pharmacokinetic profile may be useful for reducing toxicity and resistance while increasing therapeutic efficacy [25].

Liposomes are well-known drug delivery systems, already clinically approved, exploited for improving pharmacokinetics, anticancer efficacy, and reducing systemic toxicity of many anticancer drugs. The PEGylated form of liposomes, called stealth liposomes (SLs), represents a smart approach to escape macrophage uptake and gain a prolonged circulation time in blood. Recently, we proposed a new type of PEGylated liposomes in which the hydrophobic anchor, which keeps the polymer attached to the liposome surface, was greatly increased by linking up to four phospholipid moieties to a single PEG chain instead of a single phospholipid (PEG-DSPE) as is commonly used in SLs [22]. These new PEG derivatives, namely PEG-DSPE_2_ and PEG-DSPE_4_, offered an increased stability of the PEG layer around the liposomes, termed SSLs. In the development of new liposomal formulation, it is of fundamental relevance the physicochemical properties of the lipid bilayer that influence the processes of drug encapsulation and release. In particular for cisplatin, phospholipids with a higher temperature of transitions (T_m_), such as HSPC, showed a better retention of the entrapped drug [26]. Taking in mind that our scope was to develop a cisplatin delivery system for the treatment of OC through intraperitoneal injections, we focused on SSLs that can release the entrapped drug within 2-3 days. The preliminary investigation based on liposomes constituted of the unsaturated S75 phospholipid and cholesterol was unsatisfactory because cisplatin was released completely within 24 h. The unsaturated S75 phospholipid increased the fluidity and permeability of the liposome bilayer with respect to saturated lipids. To balance the increased fluidity and improve the stability of the vesicles, one-third of the S75 amount was substituted with the saturated HSPC phospholipid achieving the desired release profile. Nevertheless, such cisplatin release required the development of a lyophilized liposome formulation to avoid drug leakage during storage, which would have hampered the cytotoxicity testing and the potential future exploitation. Trehalose and sucrose were investigated as a cryoprotectant to preserve liposome size and drug encapsulation. Although both agents worked well in the preservation of vesicles morphology, sucrose had the disadvantage to reduce cisplatin activity, especially in the resistant cell line (Supplementary Figure 1), thus further confirming a role of the metabolic pathway in cisplatin resistance. Differently, the cells could not metabolize trehalose; therefore, it was selected as the cryoprotectant for all the tested liposomal formulations. The stabilization offered by the PEG layer on the liposome surface was investigated by a pharmacokinetic study in rats. The profile of cisplatin concentration in plasma for SSLs, and in particular for SSL_4_, surpasses that of conventional liposomes by showing a clear half-life prolongation and an increase in the AUC values (Table 3).

The second goal of this work was to target specific pathways correlated to cisplatin resistance in order to increase drug efficacy. Cisplatin resistance is generally considered a multi-factorial phenomenon causing, among others, changes in drug transport and accumulation, increases of DNA repair and detoxification systems as well evasion from apoptotic cell death [27]. These alterations might also play a role in the observed different internalization of the cisplatin/SSL_4_s between wild-type and resistant cell lines (Figure 6). In the last years, emerging evidence supported the involvement of cell metabolism reprogramming in resistance to cisplatin [28].

G6PDH is the first and rate-limited enzyme of the PPP, which is expressed in almost all cells. Recent studies demonstrated that G6PDH was involved in cell growth modulation and carcinogenesis. In previous studies we demonstrated that the overexpression and increased enzymatic activity of G6PDH were correlated to ovarian cisplatin-resistant phenotype cells [10], suggesting that up-regulation of G6PDH activity could be a target to counteract cisplatin resistance. In fact, the combined treatment with the G6PDH inhibitors (6-AN or DHEA) and cisplatin, exhibited a selective additive effect on cisplatin-resistant cells [10].

Present data support that SSLs of cisplatin can become a strategy to counteract cisplatin resistance especially in combination with 6-AN. A combination of cisplatin/SSL_4_ with the targeted metabolic inhibition of PPP showed a better efficacy than the cisplatin alone (Figure 7). The fact that the cisplatin/SSL_4_ had a similar or better cytotoxic effect than cisplatin when both were used in combination with 6-AN, is a promising result in view of the prolonged effect that can be achieved with a liposomal formulation of cisplatin *in vivo* and the concomitant reduced toxic effect of the drug.

## MATERIALS AND METHODS

### Materials

Non-hydrogenated soy phosphatidylcholine (S75) was a kind gift of Lipoid (Ludwigshafen, DE, Germany); hydrogenated soy phosphatidylcholine (HSPC) and 1,2-distearoyl-sn-glycero-3-phosphoethanolamine (DSPE) were purchased from NOF Corporation (Tokyo, JP). Cisplatin, 6-aminonicotinamide (6-AN), triton X-100, trehalose, sucrose, *o*-phenylenediamine (OPDA), cholesterol (Chol), trypan blue, Sulforhodamine B and all solvents and reagents were obtained from Sigma-Aldrich Co. (Milan, IT). N-(Fluorescein-5-thiocarbamoyl)-1,2-dihexadecanoyl-sn-glycero-3-phosphoethanolamine triethylammonium salt (fluorescein-DHPE) was purchased by Invitrogen (USA). PEG5k-βGlu-(DSPE)_2_ (PEG-(DSPE)_2_) and PEG5k-βGlu(βGlu)_2_-(DSPE)_4_ (PEG-(DSPE)_4_) were prepared as described previously [22].

### Cell lines

#### Human ovarian carcinoma cells

IGROV1 wild-type and the cisplatin-resistant subclone (kind gift of Dr. Giuseppe Toffoli, CRO Aviano National Cancer Institute, Italy) were grown in a cisplatin-free in Roswell Park Memorial Institute medium (RPMI 1640) supplemented with 10% fetal bovine serum (FBS), 4 mM glutamine, 100 U/ml penicillin and 100 μg/ml streptomycin, in humidified condition at 5% CO_2_ and 37°C. Cisplatin-resistant subclones (respectively IGROV1 PT; Supplementary Figure 3) were generated in Toffoli's lab according to previously standardized protocols. All reagents for cell culture were from Cambrex-Lonza (Basel, Switzerland) and FBS from Gibco, Invitrogen (Carlsbad, CA, USA). The growth kinetics are shown in Supplementary Figure 4.

### Preparation of liposomes

All liposomes were prepared by the thin layer evaporation method. Classic liposomes (CLs) were prepared with S75, HSPC, and cholesterol in a molar ratio of 63:25:12. The molar ratio composition of super stealth liposomes, SSL_2_s and SSL_4_s, were 60:24:12:4 of S75:HSPC:Chol:PEG-DSPE_2_ or PEG-DSPE_4_, respectively. When required, fluorescent-labeled liposomes were prepared by co-dissolving fluorescein-DHPE (0.6% molar with respect to S75) with the lipids. The lipid mixture, composed of about 24.5 mg of phospholipids equiv. divided between the different species according to the ratios above reported, and 2.16 mg of Chol were dissolved in 0.2 ml of chloroform. A thin lipid film was obtained by evaporation of the organic solvent under a stream of nitrogen (N_2_) and further vacuum-dried for 24 h to remove any residual organic solvent. The film was hydrated with 1 ml of cisplatin solution at 1 mg/ml in PBS and the resultant liposomal suspension was incubated at 70°C for 1 h with gentle mixing. Small unilamellar vesicles (SUV) were obtained by serial extrusion cycles through polycarbonate filters with a pore size ranging from 400 to 100 nm at 70°C using a syringe extruder Liposofast (Avestin Inc., CA). The separation of the no-encapsulated drug from the liposomes was achieved by ultrafiltration with an Amicon Ultra centrifugal tube cut-off 10 kDa (Millipore). When needed trehalose as cryoprotectant was added to the hydration solution at different concentrations (cryoprotectant: phospholipid 2:1, 5:1 or 10:1 mass ratio) and to the purification solution (cryoprotectant: phospholipid 6:1, mass ratio). Then the liposomal formulations were frozen at −80°C for 24 h and, finally, lyophilized. The liposomes were reconstituted to the initial volume by the addition of milliQ water.

### Characterization of cisplatin-loaded liposomes

#### Size characterization

The average particle size and the polydispersity index (PDI) of CLs, SSL_2_s, and SSL_4_s were measured on a dynamic light scattering (DLS) instrument Zetasizer Nano ZS (Malvern, UK). Phospholipid concentrations in liposome solutions were measured by Stewart assay, based on a colorimetric determination of inorganic phosphate.

#### Drug loading

Cisplatin concentration was determined with a spectrometric method developed by Basotra et al [29]. From a solution of liposomal cisplatin aliquots of 20 μL were withdrawn and placed in 1 mL glass test tubes. Then 100 μL of OPDA solution at 1.4 mg/mL and 200 μL of phosphate buffer 5 mM pH 6.8 were added to each solution and heated at 100°C for 10 min in order to get the formation of a light green color. The solutions were cooled to room temperature, and brought to 1mL with DMF. The UV absorption at 706 nm was measured by a UV-Vis spectrophotometer.

A solution of cisplatin in 5 mM phosphate buffer pH 6.8 at a concentration of 10 μg/mL was used to prepare a calibration curve.

#### Serum stability

An aliquot of about 20 mg in phospholipid equiv. of each cisplatin-loaded liposomes was resuspended in 2 ml of FBS/PBS (50/50) mixture and independently incubated for 24 hours at 37°C. Samples were analyzed by DLS for 24 h.

#### Drug release

1 mL of a liposome suspension was loaded into a dialysis tubes (Float-A-Lyzer G2, 100,000 MWCO) and placed in a becker filled with 500 mL of PBS and kept under stirring. The system was thermostated at 37°C. Aliquots (20 μL) were withdrawn from the dialysis tube at different time points to measure the content of cisplatin as above reported.

### Cell viability assays

#### Trypan blue exclusion assay

1×10^5^ cells were plated on 12-well plates and, following overnight incubation, were exposed to a glucose-free medium added with 1mM 2-deoxyglucose (2-DG) for 24 hours. After treatment, cells were washed, detached with 0.25% trypsin-0.2% EDTA and suspended in trypan blue at 1:1 ratio in medium solution [30]. Cells were counted using a chamber Burker hemocytometer.

#### Sulforhodamine B (SRB) test

4×10^3^ cells were plated on 96-well plates and, following overnight incubation, were exposed to different treatments according to experimental protocols. After treatments cells were fixed with trichloroacetic acid and stained with SRB as previously described [10]. The bound SRB was therefore dissolved by adding 10 mM TRIS and the absorbance was measured at 570 nm using a Victor3X multilabel plate counter (Wallac Instruments, Turku, Finland).

### Immunoblot assay

1.5×10^6^ cells (IGROV1) were plated in 100 mm cell culture dish and allowed to attach overnight. After 48 hours, cells were washed and lysed with ice-cold lysis buffer (TRIS 25 mM pH 7,4; NaCl 150 mM; IGEPAL 1%; sodium deoxycholate 1%; SDS 0,1%; EDTA 1 mM) supplemented with the protease inhibitor cocktails (Roche Molecular Biochemicals, Mannheim, Germany). Cell lysates were then centrifuged at 14000 rpm for 15 minutes at 4°C and the supernatant protein content was determined by Lowry procedure (Bio-rad DC Protein Assay, MA, USA). Equal amounts of protein (40 μg) were loaded on a 10% polyacrylamide gel and electrophoretically separated in running buffer. After electrophoresis, the proteins were blotted onto a Hybond-P PVDF membrane (Amersham Biosciences, Buckinghamshire, UK) and non-specific binding sites were blocked with a 10% skim milk solution. The membrane was therefore exposed to the elected primary antibody: anti-GLUT1 (1:2000; AbCam, Cambridge, UK) or anti-G6PDH (1:500; Santa Cruz Biotechnology, Inc., Europe) and, following overnight incubation, was washed and exposed to the HRP-conjugated anti-rabbit secondary antibody (1:3500; PerkinElmer, MA, USA). The signal was visualized with an enhanced chemoluminescent kit (Amersham Biosciences) according to the manufacturer's instructions and analyzed by Molecular Imager VersaDoc MP 4000 (Bio-Rad, Hercules, CA, USA). GLUT1 and G6PDH were normalized to calnexin (1:2000; Rabbit, Santa Cruz Biotechnology). The integrated intensities of GLUT1 and G6PDH were normalized to calnexin.

### Quantitative real-time PCR

Total mRNA was extracted as per manufacturer's instructions using a Direct-zol™ RNA MiniPrep kit (Zymo Research, Irvine, CA, USA) and measured with a NanoDrop 2000 (Thermo Fischer Scientific Inc., Waltham, MA, USA). The relative expression of each gene was determined by quantitative real-time PCR (Eco™ Illumina, Real-Time PCR system, San Diego, CA, USA) using One Step SYBR PrimeScript RT-PCR Kit (Takara Bio, Inc., Otsu, Shiga, Japan) and the primers designed as follow: G6PDH: F aagaacgtgaagctccctga R aatataggggatgggcttgg; GLUT1: F atgggcttctcgaaactggg R ccgcagtacacaccgatgat; PFKM: F gccatcagcctttgacaga R ctccaaaagtcgcatcactg; PGK1: F cagctgctgggtctgtcat, R gctggctcggctttaacc; LDHA: F tggcagccttttccttagaa R cgcttccaataacacggttt. Melt-curve analysis was used to confirm the specificity of amplification and absence of primer dimers. All genes were normalized to calnexin designed as follow: F: gaagggaagtggttgctgtg R: gatgaaggaggagcagtggt. Expression levels of the indicated genes were calculated with the ΔΔCq method [31] using the dedicated Eco™ Software v4.0.7.0. Wild-type cells were used as reference sample. Briefly, this method normalizes the expression of the target genes relative to a single reference gene; thereafter the obtained relative expressions are normalized to a reference sample. The exact calculations are adapted from Livak, *et al*. as follow: ΔCq = Cq_(Target Assay)_ - Cq_(Reference Assay)_; ΔΔCq = ΔCq_(Test Sample)_ - ΔCq_(Reference Sample)_; RQ = 2^−ΔΔCq^.

### Glucose uptake

IGROV1 glucose uptake was measured as previously described [10]. Briefly, 5×10^3^ cells (IGROV1) were plated in 96-well plate and allowed to attach overnight. After 24 hours, cells were stained for 5 minutes with the glucose analog 6-NBDG (Invitrogen, Paisley, UK) and their fluorescence (λ_ex_: 465 nm, λ_em_: 540 nm) was measured by Victor3X multilabel plate counter (Wallac Instruments, Turku, Finland).

### G6PDH activity

1.5×10^6^ cells (IGROV1) were plated in 100 mm cell culture dish and allowed to attach overnight. After 48 hours, cell samples were prepared as previously described [10]. The G6PDH activity was assayed on cell supernatant as per manufacturer's instructions using the Glucose-6-Phosphate Dehydrogenase Activity Assay Kit (Cayman Chemical Company, MI, USA). The fluorescence intensity (λex/em=540/585) was measured using a Victor3X multilabel plate counter (Wallac Instruments, Turku, Finland). The G6PDH activity (nmol/min/ml) was calculated as per manufacturer's instructions.

### Live-cells confocal microscopy

25×10^4^ cells (IGROV1) were grown in 4-wells glass-bottom culture chambers (Sarstedt AG & Co, Nümbrecht, Germany) and, after 24 hours, were labeled with LysoTracker® Deep Red (Molecular Probes, Invitrogen) or CellMask^TM^ Orange Plasma membrane Stains (Molecular Probes, Invitrogen) as per manufacturer's instructions. After staining, cells were washed, loaded with the fluorescent cisplatin/SSL_4_ 0.5 μM and the images were periodically acquired for 24 hours using a time-lapse confocal microscope (Zeiss LSM 800, 40X magnification). A volumetric reconstruction was then obtained using the software ZEN 2.1 (blue edition).

### Statistical analyses

All data were analyzed with GraphPad software and are expressed as mean ± SEM. Standard ANOVA procedures were performed for all the cell viability assays except for the SRB tests after cisplatin and 6-AN treatments that were analyzed with unpaired Student's *t*-tests. One sample *t*-tests were used to analyze results expressed as a ratio of control (qRT-PCR and glucose uptake), while unpaired Student's *t*-tests were performed for all the other results. Significance was considered at *p*< 0.05.

### Isobolographic analysis

The isobolographic analysis was used to determine the effect of cisplatin and 6-AN co-treatment. Isoboles are defined as iso-effect curves. that show drug concentrations resulting in equal effect [32, 33]. From iso-effective curves it is possible to verify the presence of simple additivity, supra-additivity (synergism) or infra-additivity (antagonism).

### Ethical statement

The study protocol was approved by the Ethics Committee of the University of Padova and the Italian Ministry of Health (938/2016-PR), and animals were handled in compliance with national (Italian) Legislative Decree 116/92 guidelines and with the “Guide for the Care and Use of Laboratory Animals” by the National Research Council of the National Academies.

### Pharmacokinetic study

FemaleLewis rats (weight 210-250 g) were purchased from Charles River Labs and housed in a temperature and humidity controlled room under a constant 12-hour light/dark cycle. Animals had free access to water and food ad libitum. Animals were randomly divided into four groups of three units each. Free and liposomal cisplatin, in CL, SSL_2_, and SSL_4_, were injected as a single bolus via tail vein at the concentration of 3 mg/kg cisplatin equiv. Blood samples (100-150 μL) were withdrawn by the tail-tip cut method at predetermined time-points and placed into heparinized Eppendorf test tubes. Plasma samples were centrifuged at 1000g × 15′ to eliminate the red blood cells. The supernatant plasma (50 μL) were stored at −80°C until the determination of cisplatin by atomic absorption spectroscopy (AAS) on a Varian AA240Z, equipped with a sample dispenser and a graphite tube atomizer, GTA120. Cisplatin concentration in serum samples was measured against a standard curve obtained with a cisplatin standard solution. Each sample was mineralized using a solution of concentrated nitric acid and hydrogen peroxide (34.5-36.5%) in 1:4 (v:v). The mineralization process comprised four stages: a drying stage consisting of 3 steps at temperature of 85, 95 and 120°C respectively, an ashing stage at 1000°C in 3 steps of 5.0, 1.0 and 2.0 s, respectively, an atomization stage at 2700°C in 3 steps of 0.9, 2.0 and 2.0 s and a stage of burning-clean with cooling down at 50°C.

The instrument was operated at a wavelength of 265.9 nm with a slit band of 0.2 nm. Each sample was analyzed in duplicate and the absorbance values were averaged.

The pharmacokinetic data elaboration was performed by PkSolver software by applying a bicompartmental model [34].

## SUPPLEMENTARY MATERIALS FIGURES AND TABLES


