# Supplementary material for: Cisplatin liposome and 6-amino nicotinamide combination to overcome drug resistance in ovarian cancer cells
Source: Oncotarget. 2018 Mar 30;9(24):16847–60. doi: 10.18632/oncotarget.24708 (PMC5908290; doi:10.18632/oncotarget.24708)
Supplement: Supplementary file 1 [file oncotarget-09-16847-s001.pdf]

Cisplatin liposome and 6-amino nicotinamide combination to overcome drug resistance in ovarian cancer cells

SUPPLEMENTARY MATERIALS

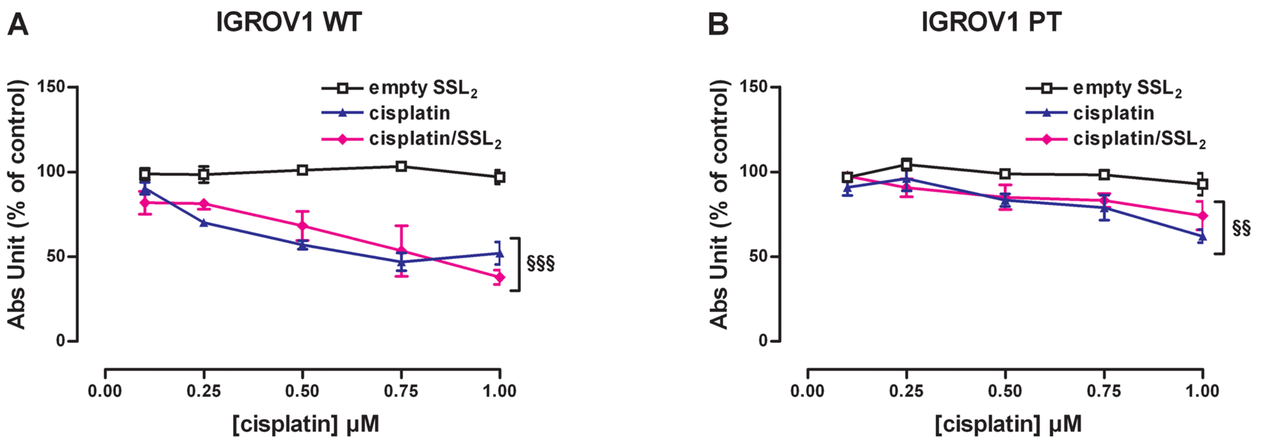

Supplementary Figure 1: Cisplatin/SSL2 delivery does not change cisplatin effectiveness in both IGROV1 cell lines. **(A-B)** Effect of cisplatin alone or delivered by SSL_2 liposomes (lyophilized with sucrose) in cisplatin-sensitive (A) and cisplatin-resistant (B) cells. The effect was measured after 24+48 h of treatments by SRB test. Data are the mean ± SEM of at least 3-4 independent cultures. §§§p<0.001, §§p<0.01; treatment *vs* control. Data are expressed as percentage of control.

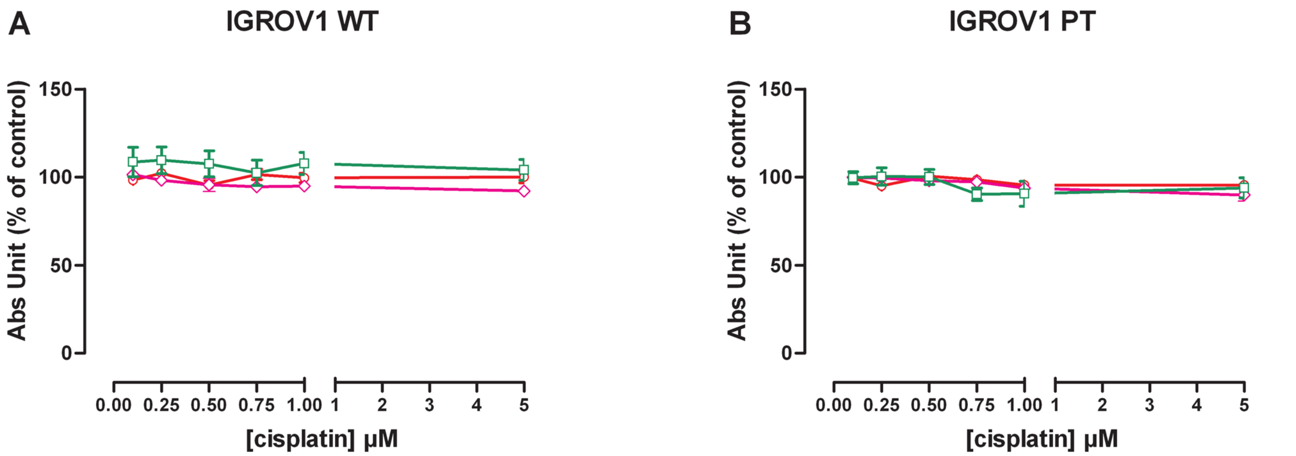

Supplementary Figure 2: Empty liposomes do not affect IGROV1 cell viability. (A-B) Effect of 3 different liposomal formulations (lyophilized with trehalose) in cisplatin-sensitive (A) and cisplatin-resistant (B) cells. The effect was measured after 24+48 h of treatments by SRB test. Data are the mean ± SEM of at least 3-4 independent cultures. Data are expressed as a percentage of control.

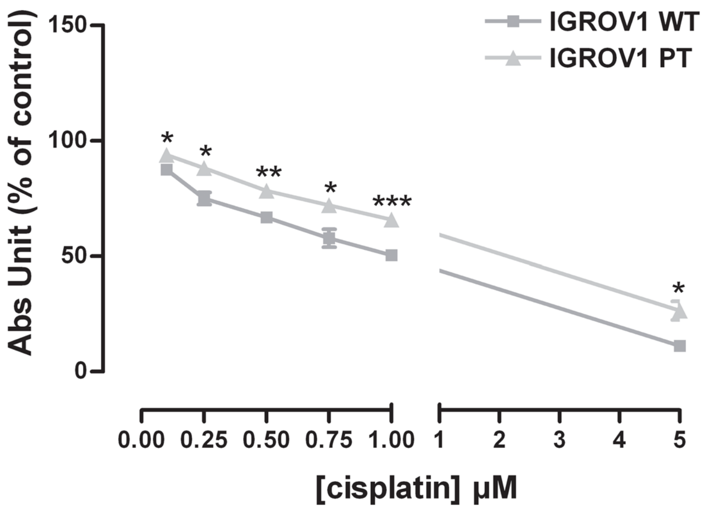

Supplementary Figure 3: Cisplatin sensitivity in IGROV1 WT and IGROV1 PT cell lines. Effect of cisplatin on IGROV1 cell viability. The effect was measured after 24+48 h of treatments by SRB test. Data are the mean ± SEM of at least 4-6 independent cultures. $^{***}p<0.001$, $^{**}p<0.01$, $^{*}p<0.05$; IGROV1 PT *vs* IGROV1 WT. Data are expressed as a percentage of control.

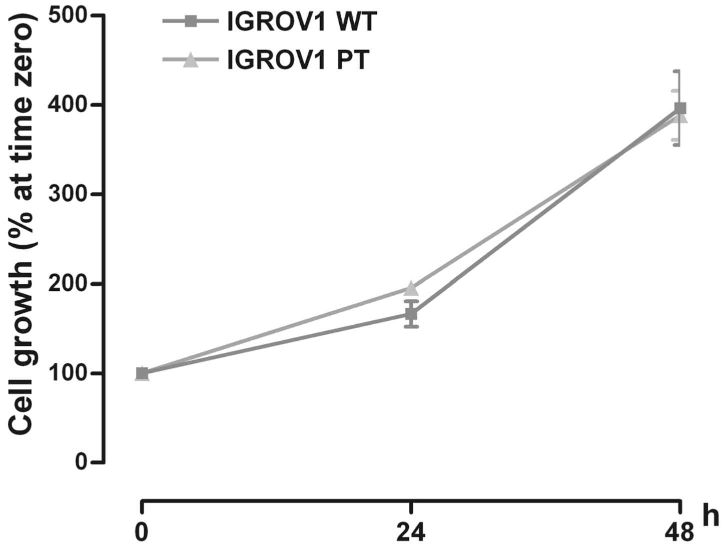

Supplementary Figure 4: Growth kinetics curves in culture conditions. Data are expressed as percentage of cell number compared to t0. Data are the mean ± SEM of 3-4 independent experiments.

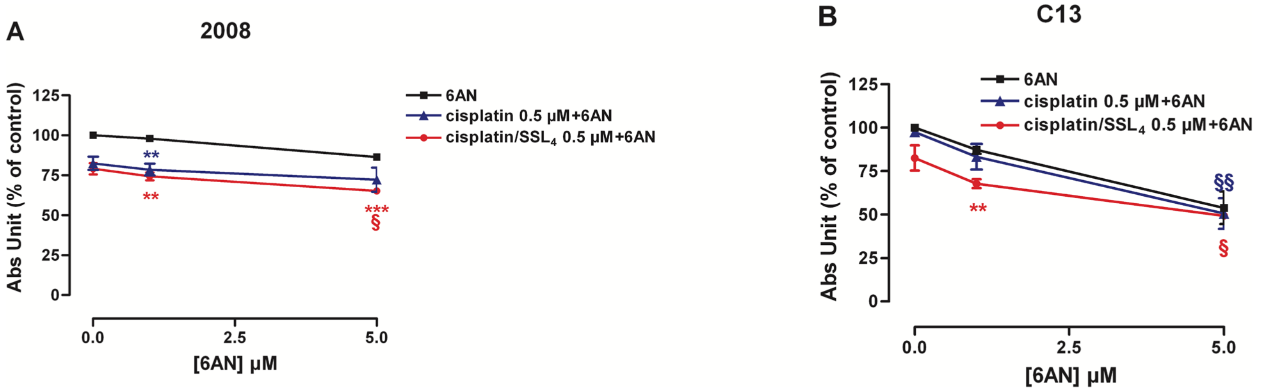

Supplementary Figure 5: Co-treatment of 6AN and cisplatin/SSL4 increases drug activity in 2008 and C13 cells. (A-B) Effect of 6AN and cisplatin/SSL_4 association in cisplatin-sensitive (A) and cisplatin-resistant (B) cells. The effect was measured after 24+48 h of treatments by SRB test. Data are the mean ± SEM of at least 3-4 independent cultures. $^{***}p<0.001$, $^{**}p<0.01$, $^{*}p<0.05$; association *vs* 6AN. §§$p<0.001$, §§$p<0.01$; association *vs* cisplatin. Data are expressed as a percentage of control.

Supplementary Table 1: Results of survival percentages for different experimental conditions of single drugs and drugs combinations

	Survival (%)	
	**IGROV1 WT**	**IGROV1 PT**
**cisplatin 0.1 µM**	89.93	93.17
**cisplatin 0.5 µM**	68.55	82.36
**cisplatin 1 µM**	53.08	67.54
**6-AN 1 µM**	85.28	97.82
**6-AN 5 µM**	86.30	97.74
**6-AN 10 µM**	80.26	32.07
**cisplatin 0.1+6-AN 1**	85.57	82.11
**cisplatin 0.1+6-AN 5**	84.57	64.92
**cisplatin 0.1+6-AN 10**	79.15	29.03
**cisplatin 0.5+6-AN 1**	62.91	78.06
**cisplatin 0.5+6-AN 5**	58.68	65.51
**cisplatin 0.5+6-AN 10**	59.27	39.11
**cisplatin 1+6-AN 1**	58.21	57.33
**cisplatin 1+6-AN 5**	60.74	54.62
**cisplatin 1+6-AN 10**	49.20	28.74

Supplementary Table 2: Combination index of cisplatin with 6-AN in IGROV1 WT. and IGROV1 PT

Conc. cisplatin (µM)	Conc. 6-AN (µM)	IGROV1 WT		IGROV1 PT	
		Effect	CI	Effect	CI
0.1	1	0.856	1.486	0.82	0.408
0.1	5	0.846	2.599	0.606	0.5
0.1	10	0.728	0.264	0.29	0.402
0.5	1	0.629	0.776	0.78	1.007
0.5	5	0.604	0.689	0.655	0,89
0.5	10	0.593	0.654	0,44	0.71
1	1	0.582	1.244	0.573	0.986
1	5	0.607	1.398	0.546	0.813
1	10	0.492	0.829	0.287	0,495

Supplementary Table 3: IC50 values of cisplatin and cisplatin/SSL4s

	IC50 (µM)	
	**cisplatin**	**cisplatin/SSL_4**
**IGROV1 WT**	2.34	2.87
**IGROV1 PT**	3.18	3.25
